# Adalimumab (Humira™) in Ophthalmology: A Review of the Literature

**DOI:** 10.4103/0974-9233.71588

**Published:** 2010

**Authors:** Piergiorgio Neri, Marta Lettieri, Cinzia Fortuna, Manuela Zucchi, Mara Manoni, Silvia Celani, Alfonso Giovannini

**Affiliations:** 1 The Eye Clinic-Ospedali Riuniti Umberto I-G.M. Lancisi-G. Salesi, Ancona, Italy; 2 Polytechnic University of Marche, Ancona, Italy

**Keywords:** Adalimumab, Immunesuppression, Macular Edema, Uveitis, Vasculitis

## Abstract

Tumor Necrosis Factor alpha (TNF-α) is a pleiotropic cytokine which plays a primary role in the induction of inflammation in autoimmune diseases. The newest anti-TNF-α agent is adalimumab (Humira, Abbott Pharmaceutical Inc.), a human-derived antibody. This review summarizes the characteristics of adalimumab, highlighting its clinical use in systemic and ocular inflammatory disorders, and the possible therapeutic strategies. Adalimumab has been successfully used for the treatment of rheumatoid arthritis, ankylosing spondylitis, and psoriasis arthritis. More recently, adalimumab has shown promising qualities in controlling intraocular inflammations, even though this has been used prevalently as a rescue therapy for unresponsive cases. This biologic agent was also used in pediatric cases, showing a good safety and efficacy profile. Albeit no direct comparison with other biologics has been done, and adalimumab seems to be equivalent to the other anti-TNF-α, the switching to adalimumab can offer a better uveitic control. Adalimumab is a promising drug for the treatment of uveitis, even though further studies are needed on its application as a primary therapy in uveitis.

## INTRODUCTION

Tumor necrosis factor alpha (TNF-α) is a pleiotropic cytokine produced by a variety of cells, comprehending T lymphocytes. TNF-α mediates its effects through two receptors, known as p55 (TNF-R1), and p75 (TNF-R2).^1^,^2^ TNF-α plays a primary role in both the induction and the maintenance of inflammation in autoimmune reactions: as soon as the inflammation begins, TNF-α acts to activate T-cells and macrophages, up-regulating the expression of endothelial adhesion molecules, and pro-inflammatory cytokines.^1^,^3^,^4^

Since noninfectious intermediate, posterior, and panuveitis^5^,^6^ are putative antigen-specific CD4 T-cell–mediated autoimmune diseases, TNF-α plays a key role in their pathogenesis:^7^–^9^ it is known that TNF-α is elevated in the aqueous humor and serum of patients affected by uveitis,^10^–^14^ playing a role similar to rheumatoid arthritis (RA).^15^

Infliximab (Remicade, Schering-Plough Pharma Inc.), a chimeric IgG monoclonal antibody, was the first commercially available drug targeting TNF-α.^16^ Etanercept (Enbrel, Whiet Pharmaceuticals Inc.),^17^ a p75 TNF-α receptor fusion protein,^17^ is no longer used in ocular inflammations since it has been proven to exacerbate the uveitis severity.^18^

In 1998, Dick *et al*.^19^ showed that TNF-α inhibition with a p55 TNF- receptor fusion protein (TNFr-Ig) lowered the intraocular inflammation. This evidence suggested that TNFr-Ig may induce the deviation of the immune response toward the Th2-type in parallel with the improvement in clinical activity.

The newest anti-TNF-α agent is adalimumab (Humira, Abbott Pharmaceuticals Inc.), a recombinant human IgG1 monoclonal antibody targeting the TNF-α; adalimumab has been recently introduced for the treatment of RA,^20^ ankylosing spondylitis (AS),^21^ and psoriasis arthritis (PsA).^22^ Adalimumab is indicated for reducing the signs and symptoms of the joint involvement of rheumatic diseases, as well as for inhibiting the progression of structural damage in adult patients with moderately-to-severely active disease.

In addition, adalimumab exhibited favorable safety and efficacy profiles while demonstrating sustained control of rheumatic diseases activity early in the course of therapy. Moreover, adalimumab has been proven effective as mono-therapy or in combination with other disease-modifying anti-rheumatic drugs (DMARDs).

Moreover, the convenient subcutaneous (SQ) dosing and administration schedule is easier for the patients compared to the other anti-TNF-α agents.

On the other hand, many questions on its use have remained unanswered and represent a challenge for the treatment strategy. Particularly, it is not clear when to initiate therapy, which agent, at what dosage, and how long the treatment should continue.^23^

In the event of failure of desired response to one biologic therapy, the efficacy of switching from infliximab to adalimumab has been described in other diseases, such as RA,^24^ showing encouraging results. This suggests to change the drugs either for poor response (primary failure) or progressive decrease of efficacy because of the production of patient antibody reaction to the non-human part of the chimeric molecule used for treatment (secondary failure).^25^

The efficacy of switching from a certain biologic agent to another has been recently proven effective,^26^ albeit additional trials are mandatory to validate the preliminary results.

In the following review, we will summarize adalimumab characteristics, highlighting the potentialities, and the possible treatment strategies, which could be applied for the therapy of ocular inflammatory diseases.

## PHARMACOLOGY

Adalimumab is human-derived antibody, produced by recombinant deoxyribonucleic acid (DNA) technology. Adalimumab tightly binds to human TNF-α, which is a naturally occurring cytokine involved in the acute phase of inflammatory immune responses. The highly specific affinity of adalimumab to TNF-α provides a blocking by interacting with the p55 and p75 cell-surface TNF receptors.^27^ Adalimumab also can lyse the surface TNF-expressing cells *in vitro* when it is the presence of complement.^27^

TNF-α seems to play a primary role in pathological inflammation and tissues damage: TNF-α is prevalent in all the tissues affected by an active inflammation, such as the synovial fluid of patients with RA or PsA, and the eye during acute uveitis.

However, the anti-TNF-α activity of adalimumab is highly specific and it does not inhibit the lymphotoxin, known as TNF-β, a cytokine produced by lymphocytes that affects a variety of cells, albeit TNF-β modulates several biological responses, resulting by the TNF-α stimulation. Specifically, these biological responses significantly influence three adhesion molecules which are responsible for leukocyte migration: endothelial leukocyte adhesion molecule (ELAM-1), vascular cell adhesion molecule (VCAM-1), and intercellular adhesion molecule (ICAM-1).

## PHARMACOKINETICS

In healthy adults, 40-mg SQ dose of adalimumab can provide a bioavailability, which is esteemed near 64%. The maximum serum concentration of adalimumab is 4.7 ± 1.6 mcg/mL, occurring within 131 ± 56 h after one 40-mg SQ dose.^21^ Recently published studies proved a concentration of adalimumab in synovial fluid ranging from 31% to 96% when compared to in the serum.

The mean terminal half-life is reported to be approximately 2 weeks, with a range of 10–20 days across studies.^27^ No gender-related pharmacokinetic differences have been observed with adalimumab after correcting for a patient’s body weight. Even if the pediatric use of adalimumab is still under investigation, there are reports in pediatrics that can suggest a certain safety of its use in such category of patients.^28^

## HUMIRA IN SYSTEMIC DISEASES

Adalimumab has been recently introduced for the treatment of RA,^20^ AS,^21^ and PsA.^22^ Published studies on adalimumab show that its use in patients with RA can be effective in providing long-term control of both the symptoms and the joint inflammation. Recently, Wiens *et al*.^29^ reported a systematic review, evaluating the efficacy and safety of adalimumab for treating RA on the basis of randomized clinical trials. The authors projected the meta-analysis to assess the efficacy, based on the changes of American College of Rheumatology (ACR) criteria and the safety, based in serious adverse events, serious infections, malignancy, and deaths. Withdrawals due to adverse events or lack of efficacy were also evaluated. The studies that were included compared the SQ doses of adalimumab 20 mg weekly or 40 mg every other week with placebo, with or without concomitant methotrexate, including only studies of moderate or high quality. The studies, that met the inclusion criteria, comprised 2,692 patients. The meta-analysis for adalimumab efficacy showed that patients treated with adalimumab had a more favourable outcome.

Regarding the safety results, no statistically significant differences were observed between the groups. The meta-analysis showed an evident efficacy of adalimumab, even though clinicians should be careful regarding the potential adverse events.

Adalimumab has been successfully used in PsA. Adalimumab has been approved for moderate-to-severe plaque-type psoriasis and psoriatic arthritis (PsA). There is evidence concerning the efficacy, clinical effectiveness, safety, and cost-effectiveness of adalimumab in the treatment of psoriasis: Schmitt and Wozel^30^ reported that adalimumab proved to be effective in moderate-to-severe plaque-type psoriasis and PsA with Psoriasis Area and Severity Index (PASI)-75 response rates of 53–80% and ACR-20 response rates of 39–58% after 12–16 weeks of treatment. In clinical practice, patients who have not responded to one TNF antagonist may respond to another one. Moreover, adalimumab has similar or better cost-effectiveness than other biologics, albeit it seems to be less efficient than methotrexate and cyclosporine in treating psoriasis and PsA.

In addition, TNF-α blockers have been shown to be very effective and safe for the treatment of a variety of sequela secondary to systemic diseases, such as RA, PsA, ankylosing spondylitis, juvenile idiopathic arthritis (JIA), and Crohn’s disease.

Although adalimumab has been prevalently used as mono-therapy^31^,^32^ and in combination with methotrexate^32^–^35^ for RA, recently, it has been reported about the successful treatment of juvenile-onset HLA-B27-associated severe and refractory heel thesitis^36^ and of orbital myositis.^37^

## HUMIRA IN OPHTHALMOLOGY

TNF-α plays a key role in uveitis. In the model of endotoxin-induced uveitis (EIU) in rats, an early elevation of TNF-α in aqueous humor and serum is detectable.^38^ In addition, intravitreal TNF-α injection in mice^39^ and rats^40^ results in acute uveitis after infiltration of polymorphonuclear granulocytes. Santos Lacomba *et al*.^10^ reported the TNF-α levels in aqueous humor and serum of patients affected by uveitis. The authors concluded that TNF-α is elevated in the serum, even though it is not relevant in aqueous humor; this immunologic pattern seems to be associated with recurrences, such as chronic uveitis.

On the basis of this observation, blocking TNF-α seems to be a promising approach in the therapy of uveitis. At this time, three drugs are commercially available to affect TNF-α expression: Etanercept, a recombinant fusion protein, combining extracellular human p75 TNF receptors with the Fc domain of a human IgG1, neutralizing TNF-α before binding to its receptor; infliximab, a mouse-human chimeric IgG1 monoclonal antibody to TNF-α, neutralizes both the soluble and the membrane-bound form of TNF-α.

Adalimumab, the newest one, is a recombinant human IgG1 monoclonal antibody, also binding soluble and the membrane-bound form of TNF-α.

In addition to the standard use in rheumatic diseases, TNF inhibitory drugs have also shown to be effective in preventing experimental uveitis.

Avunduk *et al*. used Etanercept in the endotoxin-induced model (EIU).^41^ Koizumi *et al*.,^42^ have demonstrated that Etanercept significantly reduces leukocyte rolling and adhesion in the EIU model. On the other hand, the clinical application of Etanercept in the daily practice demonstrated the attitude of such drug in inducing the uveitic process, so that this is no longer used in the uveitis patients.^18^

Various studies have demonstrated the efficacy of infliximab in Behçet’s disease and other intraocular inflammations. Mushtaq *et al*.^43^ described for the first time ever the clinical outcome of three patients with Behçet’s disease maintained on infliximab who were switched to adalimumab therapy. After adalimumab introduction, all patients remained free of recurrences with stable visual acuities. The authors hypothesized that adalimumab can maintain disease remission in Behçet’s disease.^43^

The most relevant publication has been recently published on adalimumab use in uveitis. Rudwaleit *et al*.^44^ evaluated the effect of adalimumab on the frequency of anterior uveitis (AU) flares in patients with active AS. The authors determined the history of ophthalmologist-diagnosed AU in 1250 patients with active AS who were enrolled in a multinational, open-label, uncontrolled clinical study of treatment with adalimumab, 40 mg every other week for up to 20 weeks. All AU flares were documented throughout the adalimumab treatment period plus 70 days. They compared the rates of AU flares per 100 patient years (PYs) reported during the year before adalimumab treatment with rates during adalimumab treatment, in total and by patient subgroups. The AU flare rates were significantly reduced during adalimumab treatment: the rate of AU flares was reduced by 51% in all patients, by 58% in 274 patients with a history of AU, by 68% in 106 patients with a recent history of AU, by 50% in 28 patients with symptomatic AU at baseline, and by 45% in 43 patients with chronic uveitis. In addition, the authors reported that AU flares during adalimumab treatment were predominantly mild. Only two patients with periods of high AS disease activity had new-onset AU during the treatment period. The prospective nature of this trial and the very high number of patients enrolled make very relevant the results of this study. Moreover, they suggest that adalimumab had a substantial preventive effect on AU flares in patients with active AS. On the other hand, being a noncomparative study, it is not possible to establish whether adalimumab is superior than the other biologics or this can offer better safety profile.

Another hot topic in biologics is the switching from a certain anti-TNF-α agent to another. Recently, Takase *et al*.^45^ reported their experience describing the successful switching from infliximab to adalimumab in Behçet patients. The authors considered patients that underwent treatment with infliximab, maintaining clinical remission in the patient having refractory ocular lesions to cyclosporine. All the subjects considered had an optimal control until the patients had experienced repeated infliximab-related infusion reactions. In such cases, discontinuation of the therapy led to another ocular attacks immediately. Switching to adalimumab indiced clinical remission again, suggesting that adalimumab can be a safe and effective alternative to infliximab for patients having hypersensitivity to such drug. Although the results provided for Behçet disease can be promising, no controlled trial is available and it is not possible to compare the characteristics of different anti-TNF-α, so that they are limited to refractory cases of such disease.

The same application can also be hypothesized for refractory uveitis.^46^ In a pilot study, Diaz-Llopis *et al*. assessed the efficacy and safety of adalimumab in treating refractory autoimmune uveitis. Nineteen patients were enrolled, and they received a 40-mg SQ injection of adalimumab every other week during 1 year. All patients had an active intraocular inflammation and optic coherence tomography (OCT) proved that 33 eyes (86%) had cystoid macular edema (CME) at baseline. Visual acuity improved by -0.3 log Mar in 12/38 (31%) eyes, and worsened by +0.3 log Mar in 1/38 (2.6%); 12/19 patients (63%) achieved control of their inflammation with adalimumab until the last follow-up. At the end of follow-up, there was a complete resolution of CME in 18/33 eyes (54.54%). All patients were able to reduce at least 50% of the dose of the concomitant immunosuppressive drugs at the end of follow-up. Nevertheless, adalimumab did not show serious adverse events in all patients, and only local minor side effects at the SQ injection site were observed.

**Figure 1:**
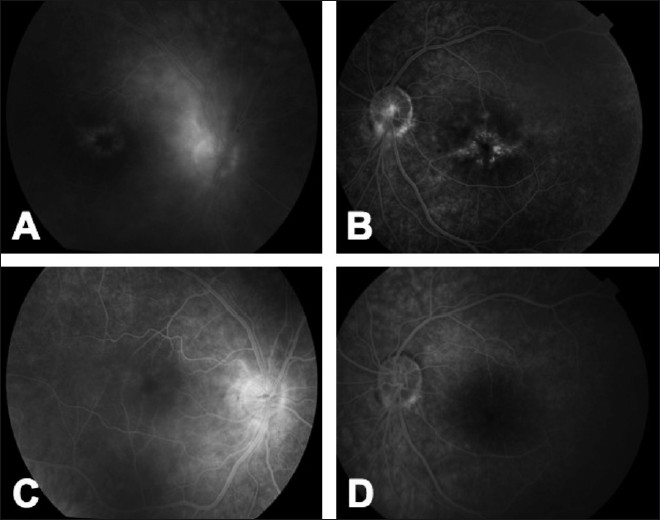
Fluorescein angiography of a patient affected by bilateral panuveitis with bilateral cystoid macular edema (A, B). The patients did not respond to the previous treatment with steroids, cyclosporine A, and azathioprine. After the introduction of adalimumab, the patient had a dramatic improvement, resolving the intraocular inflammation and the associated CME (C, D)

**Figure 2:**
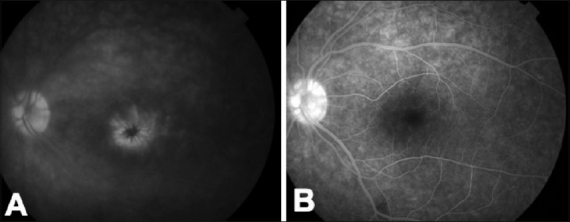
Left eye of a patient affected by panuveitis associated with CME. At baseline, the fluorescein angiography showed diffuse leakage of the retinal vessels with pooling of the dye in the foveal area, assuming the classic petaloid shape in the late phase of the angiogram (A). Adalimumab provided the re-absorption of the CME and the control of uveitis (B). No recurrence has been experienced up to the last follow-up

**Figure 3:**
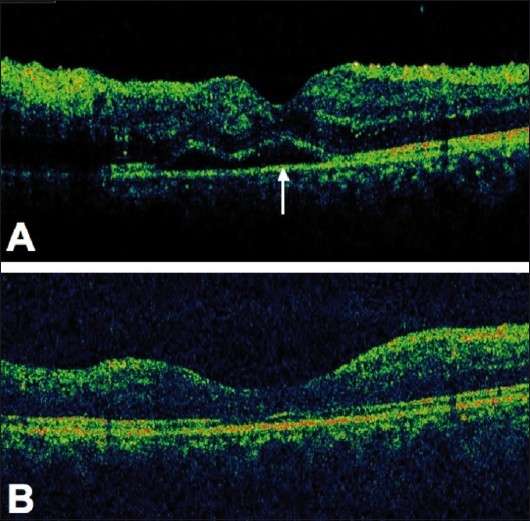
Right eye of the same patient of Figure 2. The optical coherence tomography (OCT) showed a foveal detachment (white arrow) with intraretinal edema (A). After the treatment with adalimumab the OCT had a restored retinal profile and no signs of uveitis were observed (B)

Another potential application of adalimumab in ocular inflammations can be in idiopathic panuveitis associated with CME, unresponsive to the traditional immune suppressive treatment [Figure 1]. In our center at the ocular immunology unit of the Ospedali Riuniti Umberto I-GM Lancisi-G Salesi of Ancona, Italy, we have treated successfully two patients with active bilateral idiopathic panuveitis associated with CME (unpublished data) who were unresponsive to previous treatments with traditional immune suppressive agents. At last follow-up, both patients had improved the best-corrected visual acuity (BCVA) and a fully controlled ocular inflammation [Figure 2]. In addition, the considered patients did not show signs of CME up to the last follow-up [Figure 3], which was not inferior to 9-month in both patients.

Pediatric uveitis represents another hot topic in ocular inflammations: long lasting, recurrent diseases, and especially chronic AU needs early and aggressive treatment, for resulting in good visual acuity. Because periocular and periarticular steroid therapy is difficult to apply in children, oral corticosteroids remain the first line of treatment. Side effects such as Cushing’s Syndrome and growth retardation are serious and not tolerable for a longer time in children. Therefore, a variety of other immunosuppressive agents are used in paediatric patients: methotrexate, cyclosporin A, azathioprine, cyclophosphamide and mycophenolate mofetil are used as appropriate^47^, although none of these drugs has been demonstrated to be effective in controlled studies. The difficulty in conducing randomized-controlled trials is due to several reasons: fundings are often not available for such studies and, moreover, only few uveitis centers have sufficient numbers of patients with severe, complicated disease courses, suitable for enrolment in such investigations. The aim for immunosuppressive therapies of uveitis in children is to prevent severe sequela, such as cataract and uveitic glaucoma, to reduce the recurrence rate, and to lower the toxicity of treatment.

The most relevant inflammatory disease in childhood is AU secondary to JIA with or without antinuclear antibodies, even though many other uveitis sub-types can provoke several severe sequela, such as intermediate uveitis and some other posterior uvetis.

Recently, Tynjälä *et al*.,^48^ evaluated the efficacy of adalimumab in JIA-associated uveitis, reporting that 7/20 (35%) patients had an improvement, 1/20 (5%) worsened and 12/20 (60%) did not show a significant change in the activity of uveitis. The mean number of flares/year decreased from 1.9 to 1.4 during adalimumab treatment. Serious adverse events or side effects were not significant, so that adalimumab can be considered a potential treatment option in JIA-associated uveitis, even in patients nonresponsive to previous other anti-TNF therapy. Nevertheless, seven patients in this trial discontinued adalimumab during the follow-up: six because of inefficacy and one because of inactive uveitis. These data suggest to better evaluate the role of such drug by creating randomized controlled trials, particularly for the pediatric use of adalimumab.

## CONCLUSIONS

TNF-α is a pleiotropic cytokine produced by a variety of cells that plays a primary role in uveitis pathogenesis. The effect of anti-TNF-α treatment in uveitis is explained by increasing the fraction of peripheral CD4+ T cells expressing IL-10 with a recovery of visual function.

TNF-α block causes deviation of the immune response toward the Th2 type. This has been demonstrated in patients who were treated with a recombinant protein generated by fusing the p55 TNF-α receptor with human IgG1. In addition, down regulation of macrophages has been shown to be an important mechanism for TNFα-blocking drugs.^49^–^51^

Adalimumab has been recently used for the treatment of several rheumatic diseases and represent the newest biologic agent targeting TNF-α. The application in the field of uveitis is relatively new, stimulating authors to produce an increasing number of publications. Adalimumab has been successfully used in refractory uveitis in adults,^46^ without showing any serious adverse event during the treatment. Moreover, adalimumab has less predisposition to generate allergic events. This can be explained by the biochemical structure of the molecule which is more humanized compared to infliximab. In addition, the strength of adalimumab is represented by its efficacy that seems to be comparable with infliximab, the other biologic agent widely used in uveitis, and by the SQ administration that is easier than the intravenous one. Moreover, the cost effectiveness of such drug appears to be favorable and can offer a valid option for unresponsive uveitis.

The application of adalimumab in certain aggressive types of ocular inflammation, such as Behçet disease, represent a main topic in uveitis treatment.^43^,^45^ Adalimumab has shown a more comparable efficacy than infliximab in Behçet’s disease, offering a better patient compliance due to the SQ administration. On the other hand, the limited number of trials available is limiting the use of adalimumab to those cases, which are not responding to the standard of care.

An important issue for adalimumab is represented by the pediatric use:^28^ the drug seems to be better tolerated by children and did not show significant side effects during the treatment. In addition, adalimumab has shown a more reliable efficacy in controlling the ocular involvement in patients with rheumatic diseases, suggesting that this medication can be indicated in such cases.

In conclusion, adalimumab is a promising drug for the treatment of several types of uveitis, even though further studies are needed on its application as primary treatment in ocular inflammatory diseases.
